# The p21 dependent G2 arrest of the cell cycle in epithelial tubular cells links to the early stage of renal fibrosis

**DOI:** 10.1038/s41598-019-48557-8

**Published:** 2019-08-19

**Authors:** Takayuki Koyano, Masumi Namba, Tomoe Kobayashi, Kyomi Nakakuni, Daisuke Nakano, Masaki Fukushima, Akira Nishiyama, Makoto Matsuyama

**Affiliations:** 1 Division of Molecular Genetics, Shigei Medical Research Institute, 2117 Yamada, Minami-ku, Okayama, 701-0202 Japan; 2 Shigei Medical Research Hospital, 2117 Yamada, Minami-ku, Okayama, 701-0202 Japan; 3 Department of Pharmacology, Kagawa University Medical School, 1750-1 Ikenobe, Miki-cho, Kagawa, 761-0793 Japan

**Keywords:** DNA damage and repair, Genetic techniques, Mechanisms of disease

## Abstract

Renal fibrosis is accompanied by the progression of chronic kidney disease. Despite a number of past and ongoing studies, our understanding of the underlying mechanisms remains elusive. Here we explored the progression of renal fibrosis using a mouse model of unilateral ureter obstruction. We found that in the initial stage of damage, where extracellular matrix was not yet deposited, proximal tubular cells arrested at G2 of the cell cycle. Further analyses indicated that the cyclin-dependent kinase inhibitor p21 is partially involved in the G2 arrest after the damage. A newly produced monoclonal antibody against p21 revealed that levels of p21 were sharply upregulated in response to the damage during the initial stage but dropped toward the later stage. To investigate the requirement of p21 for the progression of renal fibrosis, we constructed the novel *p21* deficient mice by *i*-GONAD method. Compared with wild-type mice, *p21* deficient mice showed exacerbation of the fibrosis. Thus we propose that during the initial stage of the renal damage, tubular cells arrest in G2 partially depending on p21, thereby safeguarding kidney functions.

## Introduction

Fibrosis is one of the prominent features of organ disorders where extracellular matrix (ECM) components accumulate excessively. Although numerous studies have sought to uncover the underlying mechanisms in animal models, the molecular details remain unclear. Renal fibrosis is associated with the progression of chronic kidney disease (CKD), for which effective treatments have not yet been developed. Myofibroblasts which mainly express ECMs and contribute to the progression of renal fibrosis are delivered from a variety of origins^1–3^. Epithelial-to-mesenchymal transition (EMT) in proximal tubular cells is one of the key processes in renal fibrosis^4^. Previous studies indicate that proximal tubular cells are transformed to interstitial fibroblasts or myofibroblasts by activation of various signaling cascades, such as the transforming growth factor-β (TGF-β) and Wnt pathway^5–9^.

A proliferative response to the damage is a hallmark of acute kidney injury (AKI)^10,11^. In principle, cell cycle progression is faithfully moderated by the activation of cyclin-dependent kinases (CDKs). CDK inhibitors downregulate the activity of cyclin-CDK complexes via binding^12^. The CDK inhibitor p21 belongs to the Cip/Kip superfamily and plays multiple roles in the regulation of not only cell cycle control but also cellular senescence^13–15^. In the kidney, *p21* mRNA is not expressed under unperturbed conditions, but induced after the damage^16^. Mice lacking *p21* show opposite phenotypes, amelioration and exacerbation, depending on the type of damage^17–19^. It has recently been reported that *p21*-deficient mice avoid liver fibrosis because of the elimination of senescent liver stellate cells^20^. Thus, the role of p21 in the progression of fibrosis appears complex and its critical role is still under debate.

To maintain genome integrity from various types of damages, such as DNA double strand breaks (DSB), cells also possess checkpoints which comprise a series of signaling pathways^21,22^. The DNA damage response (DDR) is one of the protective mechanisms against genome instability. The checkpoint kinase Chk1 that is activated upon DSB, phosphorylates various substrates, thereby inducing G2 arrest until the damages are rectified^21,23^. Activation of Chk1 is observed in the process of renal damage in rats^24^. In addition, G2/M cell cycle arrest is also induced after renal damage^8,11,25^. Under sustained damaged conditions, the G2 arrested cells produce pro-fibrogenic factors, including TGF-β and CTGF^26^, which accelerate renal fibrosis progression^8,11,26^. However, the physiological significance of the cell cycle arrest in response to initial damage is unknown.

Here we explored the progression of renal fibrosis by implementing new technical tools, the novel *p21*-deficient mice and a newly produced monoclonal antibody. Our data show that in the initial stage of damage, tubular epithelial cells are arrested in G2 of the cell cycle in the mouse model of renal fibrosis. The G2 arrest is induced prior to the DNA damage checkpoint and Wnt/β-Catenin pathway activation, partially depending on the CDK inhibitor p21. In *p21*-deficient mice, the absence of this CDK inhibitor exacerbates the progression of fibrosis. These data uncover a new insight into cell cycle arrest and p21 in the complex regulation of renal fibrosis.

## Results

### Epithelial tubular cells show a proliferative response after renal injury

**Figure 1:**
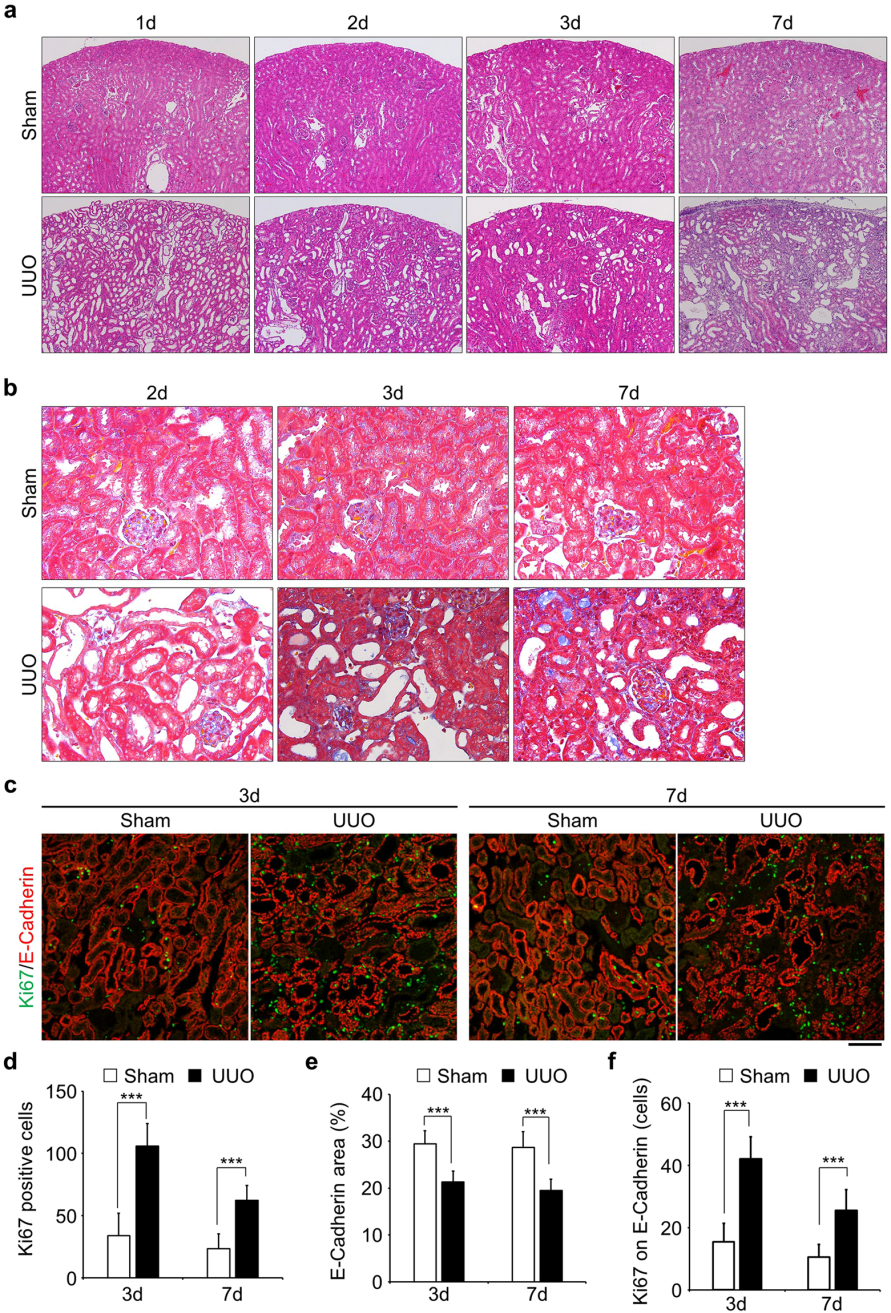
Epithelial tubular cells go into proliferative cycle upon obstruction of kidney. (**a**) Staining of Hematoxylin and Eosin (HE) of kidney slices obtained from various time points after obstruction. Sham-operated kidneys were used as a control. Images were taken at x100 magnification. (**b**) Masson’s trichrome staining. Fibrosis stained in blue. Images were taken at x400 magnification. (**c**) Co-immunostaining with Anti-Ki67 (green) and Anti-E-Cadherin (red). Ki67 was used for the identification of proliferative cells and E-Cadherin stained epithelial tubular cells. *Bar*, 100 µm. (**d** and **e**) Quantification of the number of Ki67 positive cells (d, n = 15) and E-Cadherin stained area (e, n = 15). (**f**) The number of Ki67 positive cells on E-Cadherin stained area (the number of Ki67 and E-Cadherin double stained area, n = 15). Data are as given averages ± SD (Standard Deviation); ***P < 0.001 (two-tailed unpaired *Student’s* t-test).

To analyse the multistep mechanism(s) of renal fibrosis, we conducted unilateral ureter obstruction (UUO), which is widely used as a model of renal fibrosis, and collected sham and damaged kidneys at the various time points after injury (Fig. 1a and Fig. S1). The number of glomerular was not affected noticeably in this model (Fig. 1a)^6^. In the initial stage (<3d), proximal tubules were expanded and seemed to be damaged by the obstruction and in the later stage (>3d), the area of epithelial cells was decreased and interstitial fibrosis was developed (Fig. 1b). As previously reported, proliferative cells, represented by Ki67 staining, increased after injury (Fig. 1c,d). The number of Ki67 positive cells was drastically increased in the 3 days after injury. In 7 days damaged kidney, Ki67 positive cells were increased compared with sham, but not as much as in 3 days damaged kidney (Fig. 1c,d). The area stained with antibody against E-Cadherin, a marker for epithelial cells, was also decreased by obstruction (Fig. 1e). The number of proliferative cells in epithelial tubular cells (Ki67 and E-Cadherin double positive) was increased within 3 days following damage (Fig. 1f). These data suggest that epithelial tubular cells quickly respond to the injury and re-enter the proliferative cycle from the quiescent state.

### Epithelial tubular cells arrest at the G2 phase of the cell cycle prior to renal fibrosis

**Figure 2:**
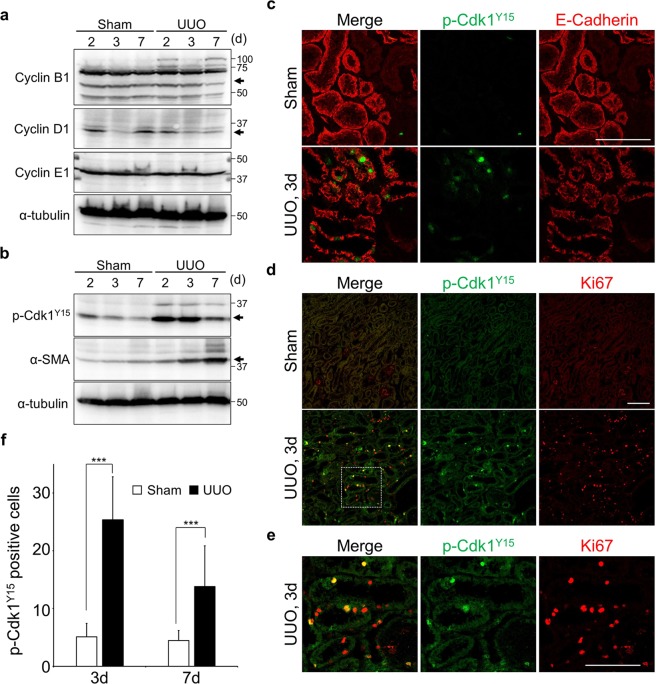
The number of the G2 cells increases prior to development of fibrosis. (**a**) Immunoblotting of tissue lysates from indicated samples. α-tubulin was used as the loading control. (**b**) Immunoblotting of tissue lysates from indicated samples. α-SMA and α-tubulin were used as markers for fibrosis and a loading control, respectively. The molecular weights (kDa) are shown in the right-hand side of the images. Arrows indicate the height of intended bands. (**c**) Co-immunostaining with Anti-p-Cdk1^Y15^ (green) and E-Cadherin (red). Scale bar, 100 µm. (**d**,**e**) Co-immunostaining with Anti-p-Cdk1^Y15^ (green) and Ki67 (red). Surrounded area is enlarged in (**e**). Scale bars, 100 µm. (**f**) Quantification of the number of p-Cdk1^Y15^ positive cells (n = 15). Data are as given averages ± SD; ***P < 0.001 (two-tailed unpaired *Student’s* t-test).

Because Ki67 stains all proliferative cells, we wondered whether epithelial tubular cells arrest at specific cell cycle stage or not. In order to elucidate which stage of cell cycle within the damaged epithelial tubular cells especially in the initial stage of damage, levels of cyclins (Cyclin B1, Cyclin D1 and Cyclin E1) were analysed (Figs 2a and S2). Unfortunately, we could not detect a clear difference between control and damaged kidneys (Figs 2a and S2). We then checked the levels of several cell cycle related proteins. From these experiments, we found that the level of phosphorylated Cdk1 (p-Cdk1^Y15^), which corresponds to an inactive form of Cdk1-cyclinB complex^27^, was dramatically elevated compared to unobstructed kidneys during the initial stage of damage (Fig. 2b). However, this elevation was transient and was reduced in the later stage (Fig. 2b, most right lane), when α-smooth muscle actin (α-SMA), a marker for fibrosis, was accumulated (Fig. 2b). Furthermore, many p-Cdk1^Y15^ positive cells were detected in the epithelial tubular cells (Fig. 2c). These data suggest that the population of tubular cells in G2 increases in the initial response to damage. Next, we undertook double staining of Ki67 and p-Cdk1^Y15^ to measure the population of G2 cells in damaged kidneys. In the control, there were very few p-Cdk1^Y15^ and Ki67 double positive cells (Fig. 2d), on the other hand, the number of co-stained cells was significantly increased after 3 days following damage (Fig. 2d–f). Compared with 3 days damaged kidney, p-Cdk1^Y15^ positive cells were decreased in 7 days damaged, however, were still significantly higher compared with control (Fig. 2f). Approximately 30% of Ki67 positive cells showed co-staining with p-Cdk1^Y15^ in 3 days damaged kidneys (Fig. S3a). In contrast, the percentage of cells positive for phosphorylated Histone H3 (p-H3^S10^), which is highly phosphorylated in mitosis^28^, was also increased but Ki67 and p-H3^S10^ double positive cells amounted to less than 10% (Fig. S3a). It should be noted that p-H3^S10^ positive cells sometimes fail show co-staining with Ki67 (Fig. S3b–e). These data suggest that epithelial tubular cells arrest prior to mitosis, namely in the G2 phase of the cell cycle, during the initial stage of damage and as such we focused subsequent experiments on the G2 arrest.

### Tubular cells arrest at G2 before activation of the DNA damage checkpoint and the Wnt/β-Catenin pathway

**Figure 3:**
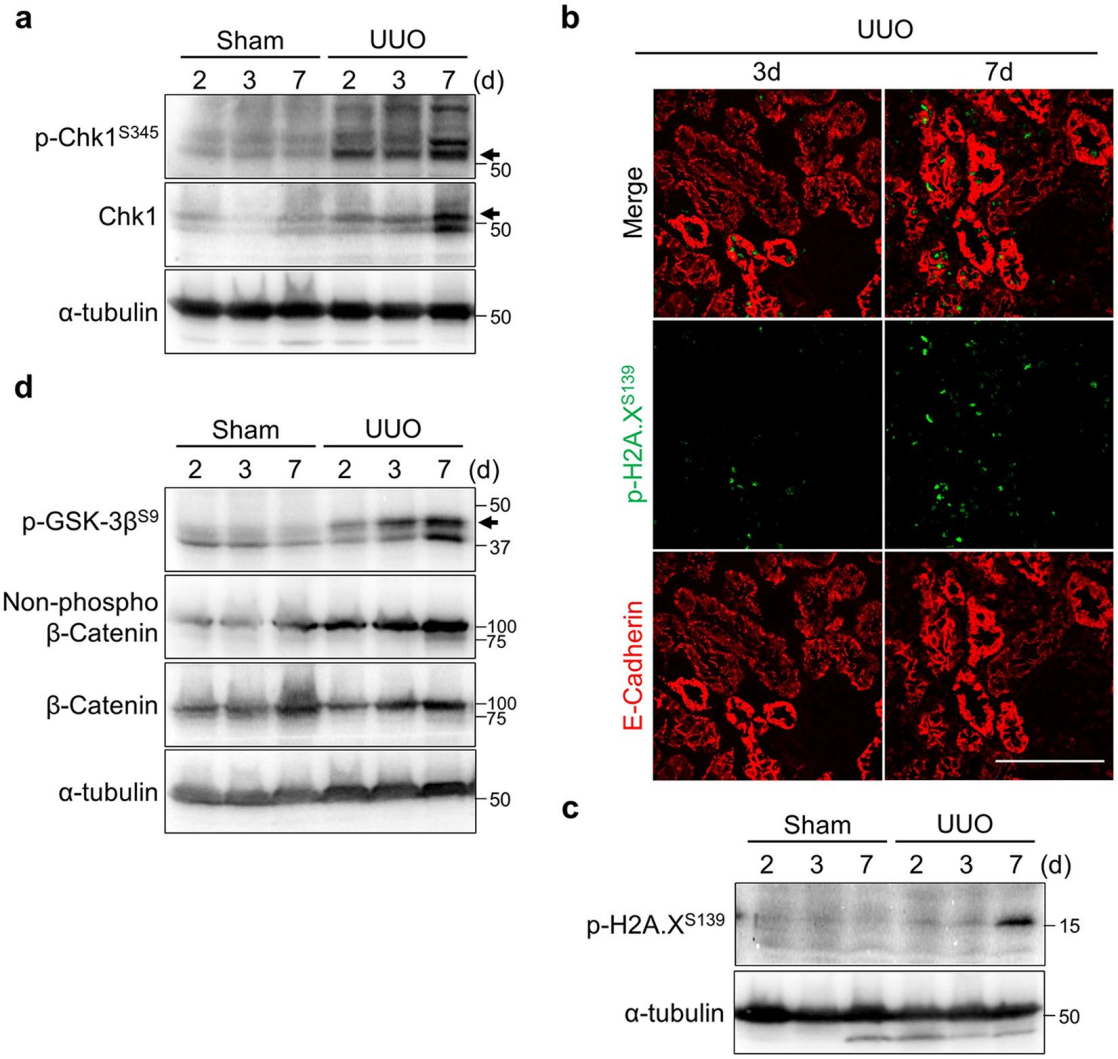
Activation of the DNA damage checkpoint and Wnt/β-catenin are induced in late stage of renal damage. (**a**) Immunoblotting with indicated antibodies of tissue lysates from indicated samples. (**b**) Co-immunostaining with Anti-p-H2A.X^S139^ (γH2A.X) (green) and E-Cadherin (red). Scale bar, 100 µm. (**c**) Immunoblotting of tissue lysates from indicated samples. Of note, γH2A.X was clearly detected 7 days after the injury in our hands. (**d**) Immunoblotting with indicated antibodies of tissue lysates from indicated samples. The molecular weights (kDa) are shown in the right side of the images. Arrows indicate the height of intended bands. Non-phosphorylated β-catenin means active form of β-catenin.

To uncover the molecular mechanisms regarding the G2 arrest during the initial stage of damage, we first examined the relationship with DNA damage checkpoint, as checkpoint activations can cause G2 arrest^21^. Additionally, activation of the checkpoint kinase Chk1 after renal ischemia/reperfusion injury (IRI) has been reported in rats^24^. Chk1 activation occurred in a time-dependent manner (Fig. 3a). The total level of Chk1 was also increased by obstruction (Fig. 3a). In agreement with this notion, the number of p-H2A.X^S139^ (γH2A.X) foci which accumulate at DNA damage loci^22^, was increased as well (Fig. 3b). This accumulation was detected more frequently at the later timepoint (Fig. 3b,c). These data suggest that the activation of the DNA damage checkpoint occurs in the later stage of renal damage.

Next, we checked the Wnt/β-Catenin cascade that induces proliferation after kidney injury^6^. Glycogen synthesis kinase-3β (GSK-3β) is implicated in the regulation of Wnt signaling via phosphorylation of β-Catenin^29^. Indeed, the phosphorylation of GSK-3β at S9, which is the negative form of GSK-3β, and the non-phosphorylated β-Catenin, which is the active form of β-Catenin were both increased accompanied with the development of fibrosis (Fig. 3d). Both the Chk1-dependent DNA damage checkpoint and Wnt/β-Catenin pathways were fully activated toward the later stage of renal damage, whereas p-Cdk1^Y15^ level was quickly elevated after the damage (Fig. 2b). Considering these data, we propose that neither the DNA damage checkpoint nor Wnt/β-Catenin signaling is responsible for the G2 arrest in the initial stage of renal damage; instead, these events were induced as a consequence of the G2 cell cycle arrest or redundant regulatory mechanisms.

### *p21* is upregulated in response to the initial stage of renal damage

**Figure 4:**
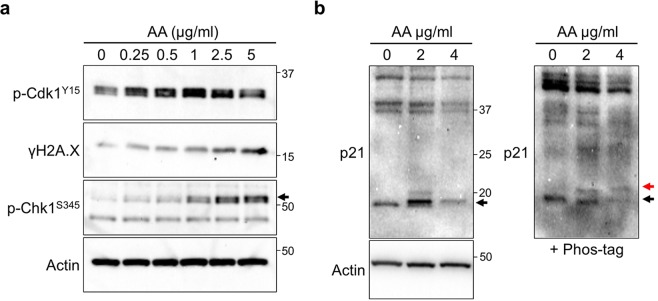
p21 is involved in the response to the damage *in vitro*. (**a**) Immunoblotting with indicated antibodies of lysates from Aristolochic Acids (AA) treated HK-2 cells. AA was added into medium and cells were sampled after 24 h. Arrows indicate the height of intended bands. (**b**) Immunoblotting with anti-p21 of AA treated HK-2 cell lysates. Samples were separated in the presence (right) or absence (left) of 25 µM Phos-tag acrylamide containing SDS-PAGE gel. In the Phos-tag containing gel, the red and black arrows indicate the phosphorylated or non-phosphorylated p21, respectively.

To explore the reason for an increased population of G2 cells, we sought the critical molecule(s) using cultured human tubular epithelial cells (HK-2). To this end, we added aristolochic acid (AA) that induces aristolochic nephropathy^11^. DNA damage and checkpoint activation were induced in a dose-dependent manner, mimicking *in vivo* damage (Fig. 4a). Intriguingly, p-Cdk1^Y15^ level was elevated at low concentrations of AA and slightly decreased at high concentration even *in vitro* (Fig. 4a). Because CDK inhibitors are involved in the regulation of cell cycle progression^12^, we examined the effect of CDK inhibitors on AA treated conditions. We found that expression of the CDK inhibitor p21 was also induced by treatment with AA and the protein level was reduced at high concentration *in vitro* (Fig. 4b). Previous work indicates that p21 is upregulated by low levels of DNA damage but is transiently degraded in response to high doses of damage, in which this degradation is induced by phosphorylation performed by Chk1^30^. In order to analyse whether p21 is phosphorylated or not, we used Phos-tag containing SDS-PAGE gel^31,32^, indeed, p21 showed double bands (Fig. 4b, right-hand side). In addition, the intensity of the p21 band was decreased at high AA concentration (Fig. 4b). These data suggest that p21 is upregulated and degraded in response to damage in cultured cells. We note that another Cip/kip superfamily CDK inhibitor, p27 was also upregulated by UUO, however, the protein level was highest after 7 days since kidney damage (Fig. S4). We consider that p27 is involved in the regulation of fibrosis but is not a critical molecule for the G2 arrest in the initial stage.

**Figure 5:**
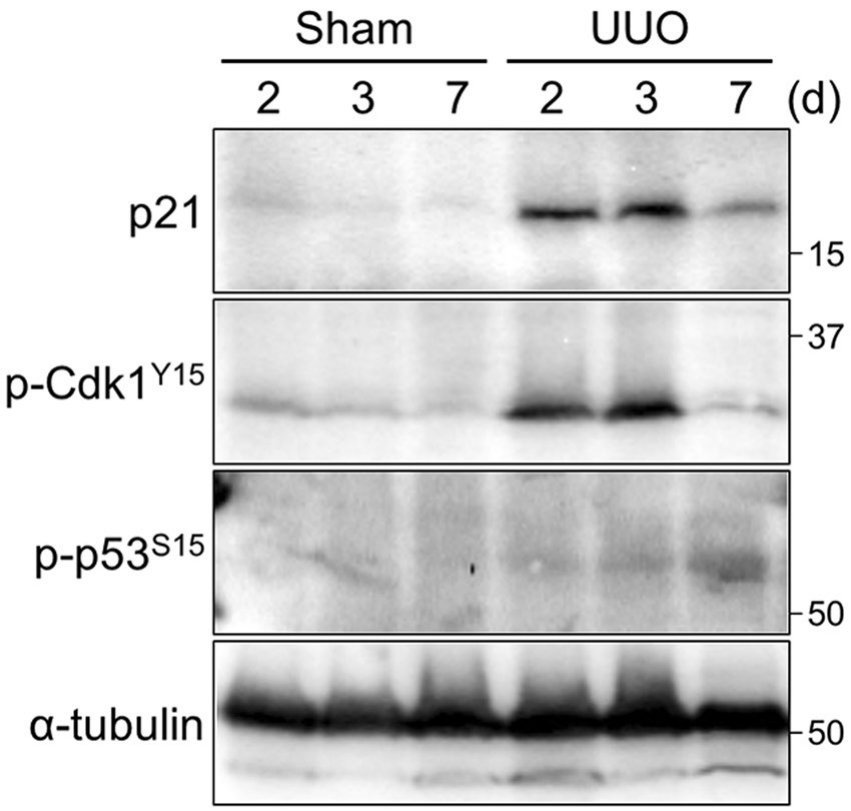
p21 is involved in the response to renal damage. Immunoblotting with a monoclonal p21 antibody of tissue lysates from indicated samples. The molecular weights (kDa) are shown in the right-hand side of the images.

These data prompted us to examine the relationship between p21 and renal fibrosis. The expression level of *p21* mRNA is very low in the control, but is slightly increased in the damaged kidney (Fig. S5). To evaluate the level of p21 protein after damage, we firstly constructed our own monoclonal antibody as the specificity of commercially available antibody is very low (Fig. S6). The newly constructed monoclonal antibody was specific to mouse p21 *in vitro* (Fig. S6b); it did not react anything in the *p21* deficient mice (See below). Furthermore, in healthy kidneys the p21 band reacting with this antibody was very low level; by contrast, however, it was sharply upregulated by the obstruction (Fig. 5). Intriguingly, the level of p21 was also reduced at day 7 damages when p-Cdk1^Y15^ level was decreased (Fig. 5); in other words, the levels of p21 and p-Cdk1^Y15^ mirrored each other.

The p21 is known as a major target of tumor suppressor p53 and is activated upon DNA damage^13,14^. Although p21 was upregulated during the initial stage of damage, phosphorylation of p53 at S15, which is induced following DNA damage^33^, was detected only at the later stage (Figs 5 and S7). These data suggest that the upregulation of p21 during the initial stage after damage is independent of p53.

### The impact of *p21*-deficiency in renal fibrosis progression

To investigate the impact of p21 in renal fibrosis, we constructed novel *p21* deficient mice (*p21*^−/−^). To this end, we took advantages of the *i*-GONAD (*i*mproved-*G*enome-editing via *o*viductal *n*ucleic *a*cid *d*elivery) method, in which handling the fertilized egg *ex vivo* is not required^34,35^. Tandem STOP codons were integrated into 60 bases after the first ATG (Fig. S8a). Correct integration was confirmed by both PCR and genome sequencing (Fig. S8c,c). Accordingly, the p21 protein was not detected even after UUO in a homozygous deletion (Fig. S8D). These data indicate that the *p21* gene was functionally deleted, similar to a reports previously described^36^.

**Figure 6:**
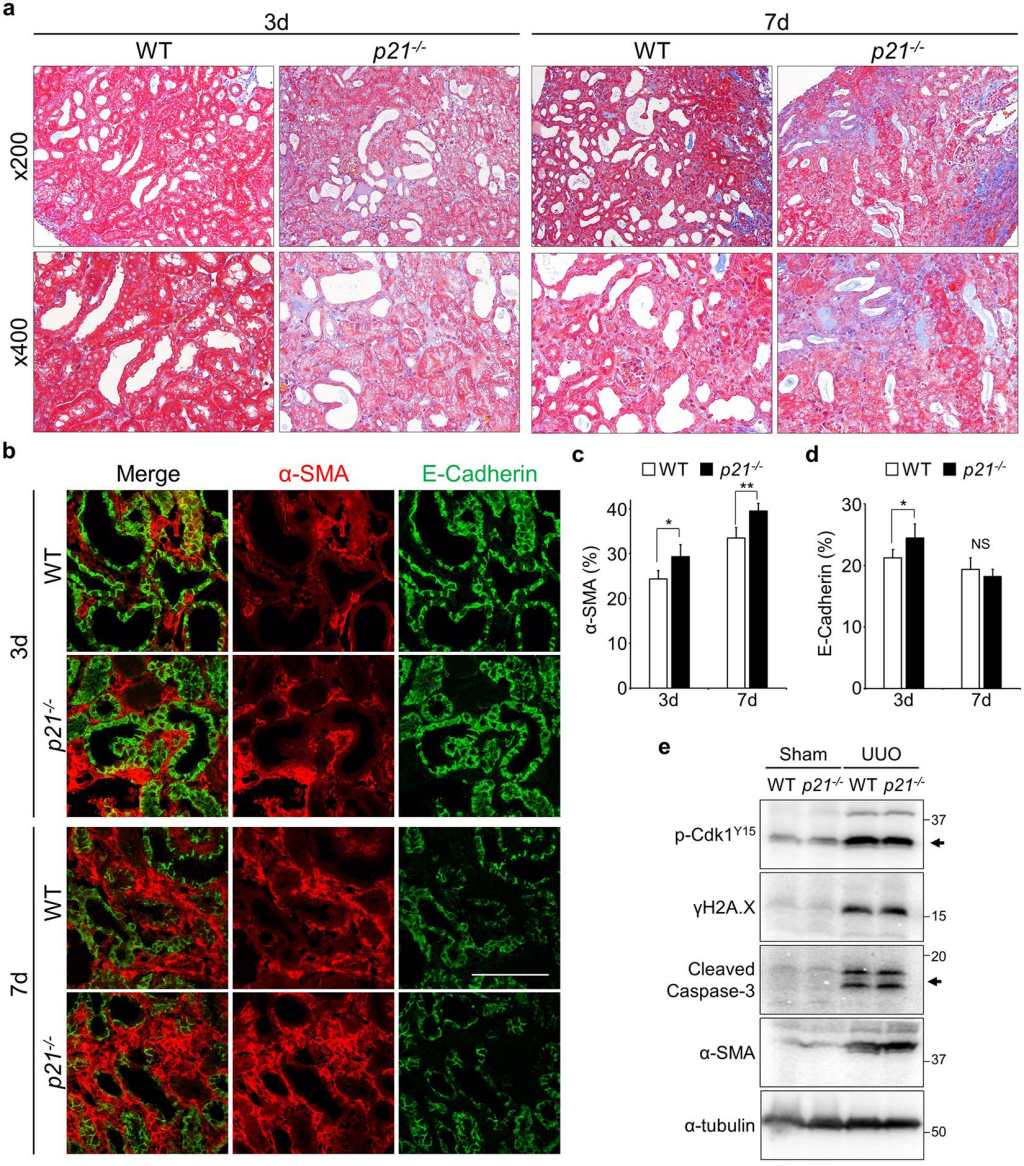
Impact of p21 on renal fibrosis progression. (**a**) Masson’s trichrome staining of tissues from WT and *p21*^−/−^ mice. Upper panels are low magnifications (x200) and lower panels were high magnifications (x400). (**b**) Immunostaining staining of obstructed kidneys in WT and *p21*^−/−^ mice. Scale bar, 100 µm. (**c**,**d**) The quantification of α-SMA (c) and E-Cadherin (d). N = 3 different mice for each time points in each genotypes. Data are as given averages ± SD; *P < 0.05; **P < 0.01. NS, Not Significant (two-tailed unpaired *Student’s* t-test). (**e**) Immunoblotting with indicted antibodies of tissue lysates from 7 days after the injury.

**Figure 7:**
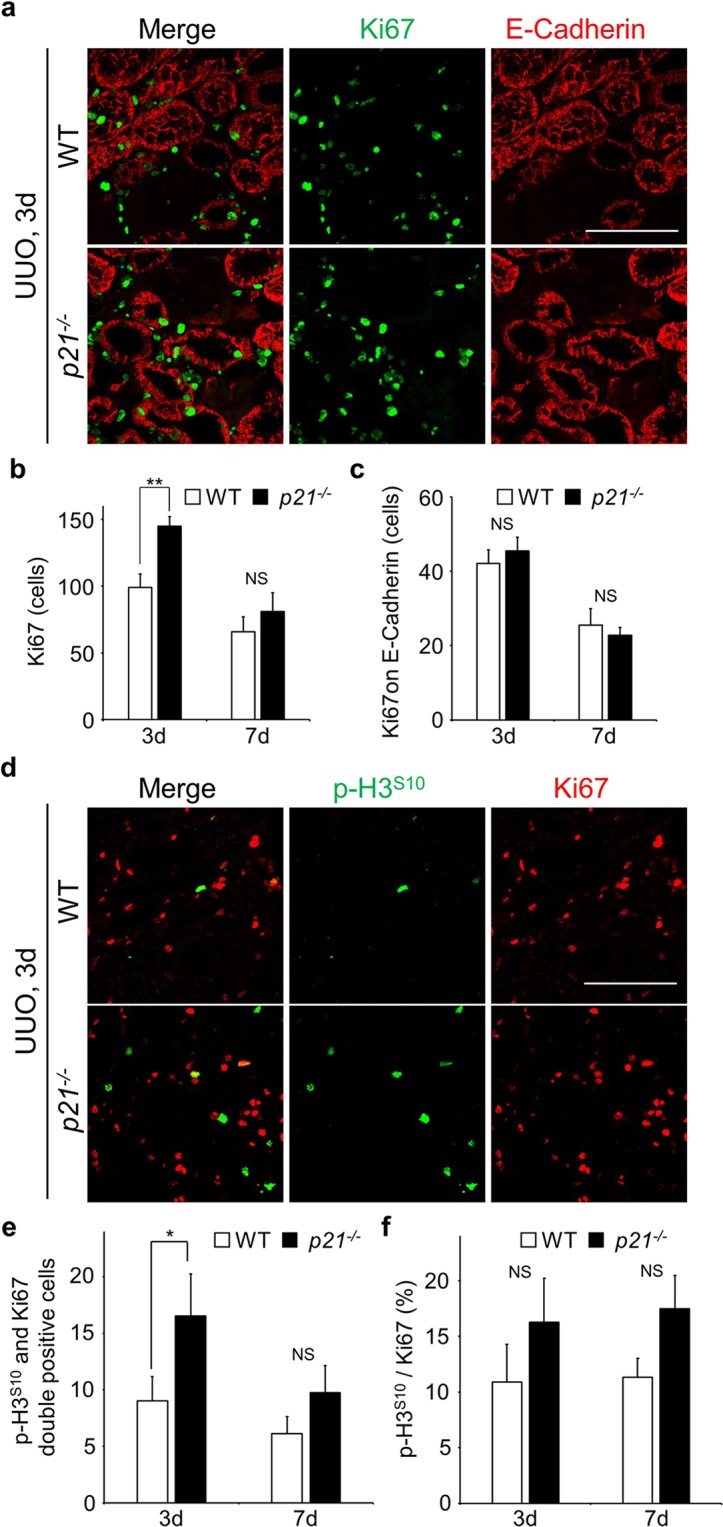
The proliferative cells are increased in *p21* deficient mice. (**a**) Co-immunostaining with Anti-Ki67 (green) and Anti-E-Cadherin (red). Scale bar, 100 µm. (**b**) Quantification of the number of Ki67 positive cells. (**c**) The number of Ki67 positive cells on E-Cadherin stained area (Ki67 and E-Cadherin double stained cells). N = 3 different mice for each time points in each genotypes. (**d**) Co-immunostaining with Anti-p-H3^S10^ (green) and Anti-Ki67 (red). Scale bar, 100 µm. (**e**) The number of p-H3^S10^ and Ki67 double positive cells. (**f**) The ratio of p-H3^S10^ per Ki67. Values were calculated by dividing the number of p-H3^S10^ and Ki67 double positive cells by the number of Ki67. N = 3 different mice for each time points in each genotypes. Data are as given averages ± SD; *P < 0.05; **P < 0.01; NS, Not Significant (two-tailed unpaired *Student’s* t-test).

Having constructed *p21*-null mice, we then conducted UUO on both wild-type (WT) and *p21*^−/−^ mice, and sampled kidneys at various time points (Fig. S9). Masson’s trichrome staining revealed that fibrosis was more progressive in *p21*^−/−^ compared to WT mice (Fig. 6a). The staining area of α-SMA in interstices was more expanded in *p21*^−/−^ (Fig. 6b–d). These data suggest that global deficiency of *p21* in mice exacerbated the renal fibrosis progression. We undertook further analyses of renal fibrosis progression in *p21*^−/−^, though the degree of DNA damage (γH2A.X) and the frequency of apoptotic cells (Cleaved Caspase-3) are not altered (Fig. 6e). Double immunostaining of Ki67 and E-Cadherin uncovered that proliferative cells were increased by *p21* deficiency (Fig. 7a,b). Furthermore, the number of Ki67 positive epithelial cells was not largely changed (Fig. 7c). Interestingly, the number of p-H3^S10^ positive cells was increased in *p21* deficient mice, suggesting that mitotic cells were increased (Figs 7d–f and S10). These data suggest that the G2 arrest during the initial stage of renal damage is partially dependent on p21 and that p21 plays some protective role(s) in the epithelial tubular cells in the early stage of damage.

## Discussion

How fibrosis progresses in kidneys is a fundamental question for health and disease. Here, we addressed this question by implementing two experimental techniques in a mouse model of renal fibrosis: *i*-GONAD to produce the gene modified mice and monoclonal antibody production. Our data showed that epithelial tubular cells were arrested at the G2 phase of the cell cycle in response to damage in the initial stage. This G2 cell cycle arrest was partially caused by the CDK inhibitor p21. Furthermore, p21 activation and cell cycle arrest in the initial stage of damage seemed to depend on neither DNA damage checkpoint nor Wnt/β-Catenin. p21 deficiency exacerbated renal fibrosis compared with wild-type mice. Thus, we propose that the p21 dependent cell cycle arrest in the initial stage of damage links to the progression of the renal fibrosis.

Our results indicate that the G2 cell cycle arrest is induced even in the very early stage of damage when fibrosis is not fully developed. Several studies have indicated that the G2 arrest in proximal tubular cells after kidney injury contributes to the progression of renal fibrosis^8,11,26^. We showed that the level of the G2 arrest was inversely proportional to the progression of fibrosis. One possibility for this is that G2 arrest in acute stage may have a protective role(s) in response to the damage, but in the chronic phase or under sustained damaged, the arrest contributes to the progression of fibrosis via the production of profibrotic growth factors^26,37^. Intriguingly, in the reversible UUO model, kidney function can be recovered when the obstruction is removed within 2 days^38^. In any case, the level of the G2 arrest in proximal tubular cells is one of the features of early stage of renal damage, and is thus a good candidate of biomarker to predict fibrosis in initial stage of damage.

We showed that p21 is involved in the G2 cell cycle arrest in the initial stage of renal damage. Our constructed monoclonal antibody detected upregulation of p21 protein in the damaged kidneys. Unfortunately the p21 monoclonal antibody could not be used for immunostaining. Which cell(s) and/or region(s) express p21 after injury is one of the intriguing questions to be explored. The expression pattern of proteins is very similar to p-Cdk1^Y15^. We suppose that the epithelial tubular cells express p21 and subsequently induce the G2 arrest after injury to prevent renal fibrosis progression.

Consistent with previous studies which the level of *p21* mRNA is increased after kidney injury even in p53 null mice^16^, the upregulation of p21 during the initial damage stage appears to be independent of p53 in the regulation of renal fibrosis. We further showed that the protein level of p21 in the later stage is reduced compared with the early stage of damage, although the mRNA is expressed. The relationship between p53 and p21 is well established in cancer^13,14^. It is a very intriguing question to decipher the molecular mechanism(s) of p53-independent upregulation of p21 in the regulation of renal fibrosis. Previous work showed that the regulation of p21 is bimodal and its level is increased in response to a low dose of DNA damage but transiently degraded upon the higher levels of DNA damage^30^. The functions of p21 are regulated by multiple phosphorylation events carried out by various kinases including the checkpoint kinase Chk1^13^. We speculate that p21 is upregulated in response to renal damage during the initial stage to protect epithelial tubular cells; however, sustained renal damage causes DNA damage, leading to Chk1-dependent phosphorylation and degradation of p21. Further analysis will clear complex molecular mechanisms of p21 regulation.

Previous reports indicate that *p21*-deficiency ameliorates the development of the fibrosis and the progression of chronic renal failure after partial renal ablation^17^. On the other hand, p21 has a protective and beneficial role(s) in renal ischemia/reperfusion injury (IRI)^18,19^. In our study, p21 is initially activated and subsequently degraded in the later stage. And *p21*-deficient mice show severe fibrotic features. It seems that p21 has a protective role(s) in the progression of renal fibrosis. Nonetheless, it is possible that p21 deficiency is beneficial for fibrosis. In a recent study, *p21* knockout mice exhibited improved liver fibrosis due to elimination of senescent liver stellate cells^20^. These paradoxical functions could be attributed to different cell types and the dose of the damage used. In the kidney, a fibrotic injury-induced EMT program in epithelial tubular cells induces a G2 arrest partially dependent on p21^8^. We expect that p21 has bimodal roles, protecting or accelerating the progression of fibrosis, depending on stages of the damage (acute or chronic). Conditional p21 knockout mice, such as cell type-specific or drug inducible, will be useful for understanding these issues. Further analyses will illuminate the role(s) of p21 in the regulation of fibrosis.

Genome manipulation is a powerful tool for understanding molecular mechanisms in the fields of life science and medical research. Recent advances in genome-editing technology have enabled rapid generation of genome-edited animals with ease. However, the procedure for producing such animals involves multiple complicated steps and also necessitates skillfull techniques, *e*.*g*. handling zygotes *ex vivo*. The fact that this is time-consuming is also one of the limitations when we wish to perform genetic analysis in animals. In this study, we generated new *p21*-deficient mice by using the *i*-GONAD method^35^, though mice lacking p21 have already existed^36^. Our constructed mice showed no production of the protein even after injury. Thus, the *i*-GONAD method will enable us to analyse the phenotypes of knockout mice rapidly and efficiently in the field of animal research.

## Methods

### Mice

C57BL/6 male mice were used throughout this study. *p21*-deficient mice were generated by the *i*-GONAD method as previously described^35^. In brief, single stranded DNA containing 3 STOP codons and homology sequence against the second exon of *Cdkn1a* (cggtcccgtggacagtgagcagttgcgccgtgattgcgattgactagctagaattcccgggcgctcatggcgggctgtctccaggaggcccgagaacggt) was injected to the oviductal lumen of pregnant female mice (E0.7) with Alt-R™ CRISPR-Cas9 system (Integrated DNA Technologies) and subsequently electroporated by the electroporator (NEPA21, Neppa Gene). Guide RNA was designed using CHOPCHOP (http://chopchop.cbu.uib.no/). All materials and equipment were used in accordance with manufacture’s procedures. Correct integration was confirmed by PCR and genome sequence of pups (Fig. S8). The *p21*-deficient mice were maintained in C57BL/6 background. The male homo deletions (*p21*^−/−^) were obtained from crossing hetero female and male mice.

Renal fibrosis was induced by unilateral ureter obstruction (UUO). Left ureters of age matched male mice were surgically ligated by the 9 mm of string and collected obstructed and unobstructed kidneys at the indicated time points after the operation (Fig. S1). The sampling time points after UUO are indicated in figures. Unobstructed kidneys (right kidneys) were used as controls (sham). All animal experiments were approved by the Shigei Medical Institute Animal Care committee (permission number: #17001). Animals were handled strictly in accordance with the appropriate guidelines and regulations.

### Antibodies

The rat-monoclonal antibody against mouse p21 was constructed as previously described^39^. Essence is following. Full length of mouse p21 (mp21) was amplified from the cDNA (MMM1013-202761927, Dharmacon) with the following primers (Fw: ccgGAATTCatgtccaatcctggtgat, Rv: cgcGTCGACtcagggttttctcttgca). The amplified fragment was inserted into a pET28a vector with restriction enzymes (*Sal*1 and *Eco*R1). His-tagged full length of mp21 was expressed in BL21-CodonPlus-RP cells. Because His-mp21 was insoluble (Fig. S6A), we purified the inclusion bodies from the insoluble fraction with 8 M of Urea. Purified His-mp21 with a complete adjuvant was injected into female rats. 17 days after the injection, lymphocytes were collected from iliac lymph nodes and hybridomas made^40^. The culture supernatants of hybridomas were used as antibodies. The candidates were screened by ELISA against His-mp21 and immunoblotting against the cell lysates of Myc-mp21 expressed cos-7 cells. The mono clone was obtained by limited dilution. Other commercial antibodies used are listed in supplementary Table S1.

### Histological analyses

Kidneys were soaked in 10% buffered neutral formalin at least overnight. Subsequently, the fixed kidneys were embedded in paraffin and sliced at 3 µm thickness. Slices were stained with Hematoxylin and Eosin or Masson’s trichrome.

### Immunofluorescence staining

Frozen kidney mounted in compound were sliced at 5 µm thickness. Slices were fixed with 4% paraformaldehyde at room temperature for 10 min. After blocking in 5% donkey serum for 30 min at room temperature, slices were incubated with primary antibody, followed by Alexa Fluor labeled secondary antibodies (Molecular probes). Images were captured by confocal microscopy (FV2000, Olympus) and were processed by Olympus fluoview ver.4.0. Quantified images were taken by using 20x objective lens (512 × 512 pixel image) and analysed using ImageJ. Numbers in figures were per 512 × 512 pixel images. Higher magnification images were taken by using 60x objective lens with immersion oil (Olympus).

### Immunoblotting

Obtained kidneys were homogenized in buffer (50 mM Tris, 150 mM NaCl, 1 mM EDTA, 1% TritonX100) containing protease inhibitor (Nacalai tesque) on ice and lysates were cleared by centrifugation at 15,000 rpm for 15 min at 4 °C. Equal volume of 2xSDS-PAGE sample buffer was added to cleared sample and boiled for 5 min. Samples were stored at −80 °C. Cultured HK-2 cells were directly homogenized in a 2xSDS-PAGE sample buffer. Each sample was separated by SDS-PAGE gel (12.5%) and analysed by western blotting with appropriate 1st and 2nd antibodies. For the detection of p21 phosphorylation, phosphate affinity SDS-PAGE was carried out using 25 µM of Phos-tag acrylamide (NARD Institute)^41^. Representative blotting images of each sample are shown in figures. The original scan images are shown in supplementary information (Fig. S11). The figures are grouped from different gels, but identical samples are applied into each gels equally.

### Cell culture

Human tubular cells HK-2 were obtained from ATCC. HK-2 cells were cultured in keratinocyte serum free medium (K-SFM, Gibco). Cells were treated with the indicated concentrations of Aristolochic Acid (AA, Sigma-Aldrich). Dimethyl sulfoxide (DMSO, Wako) was used as a solvent.

### Statistical analysis

To evaluate comparisons between WT and *p21* deficient mice, three different mice of each genotypes were used. Five different regions per mouse were analysed and the averages were used as a value for the mouse. All statistical analyses were carried out using two-tailed unpaired *Student’s* t-test. P values were indicated by asterisk (*P < 0.05, **P < 0.01, ***P < 0.001). NS means “Not Significant”. P < 0.05 was considered as statistically significant.

## Supplementary information

Supplementary information
