# Supplementary material for: The p21 dependent G2 arrest of the cell cycle in epithelial tubular cells links to the early stage of renal fibrosis
Source: Sci Rep. 2019 Aug 19;9:12059. doi: 10.1038/s41598-019-48557-8 (PMC6700145; doi:10.1038/s41598-019-48557-8)
Supplement: Supplementary file 1 — Supplementary information [file 41598_2019_48557_MOESM1_ESM.pdf]

Supplementary information

The p21 dependent G2 arrest of the cell cycle in epithelial tubular cells links to the early stage of renal fibrosis

Takayuki Koyano, Masumi Namba, Tomoe Kobayashi, Kyomi Nakakuni, Daisuke Nakano, Masaki Fukushima, Akira Nishiyama and Makoto Matsuyama

Contents

Table S1: Commercial antibodies used in this study
Figure S1: Schematic outline of unilateral ureter obstruction (UUO)
Figure S2: Protein levels of CyclinB1 and CyclinD1
Figure S3: The image of double staining with p-H3^{S10} and Ki67
Figure S4: Protein levels of p27 after the injury
Figure S5: Expression level of p21 in mice after the injury
Figure S6: Construction of the monoclonal antibody against mp21
Figure S7: Levels of p-p53^{S15}
Figure S8: Construction of the *p21* deficiency mice
Figure S9: Histological staining of kidneys after UUO in *p21* deficiency mice
Figure S10: The number of p-H3S10 was slightly increased in *p21* deficient mice
Figure S11: Full scan of images for immunoblotting

Table S1: Antibodies used in this study

	Company	Clone / Catalog#	Host	Application
Ki67	abcam	SP6(ab16667)	Rabbit	IF (1/400)
Ki67	DakoCytomation	TEC3(M7249)	Rat	IF (1/200)
E-Cadherin	abcam	DECMA-1(ab11512)	Rat	IF (1/800)
p-CDK1^{Y15}	abcam	ERP7875(ab133463)	Rabbit	IF (1/200), WB(1/4,000)
p-Histone H3^{S10}	Cell signaling	D2C8(#3377)	Rabbit	IF (1/200)
p-GSK3^{S9}	Cell signaling	D85E12(#5558)	Rabbit	WB(1/2,000)
p-Chk1^{S345}	Cell signaling	133D3(#2348)	Rabbit	WB(1/1,000)
Chk1	MBL	DCS310(K0086-3)	Mouse	WB(1/1,000)
Activated β-Catenin	Cell signaling	D13A1(#8814)	Rabbit	WB(1/2,000)
β-Catenin	BD Biosciences	610153	Mouse	WB(1/10,000)
α-tubulin	Sigma	DM1A(T9026)	Mouse	WB(1/40,000)
α-SMA	Cell Signaling	1A4(#48938)	Mouse	IF (1/500), WB(1/10,000)
p-H2A.X^{S139}	Cell signaling	20E3(#9718)	Rabbit	IF (1/500), WB(1/1,000)
p21	Cell signaling	12D1(#2947)	Rabbit	WB(1/2,000)
p27	abcam	SX53G8(ab193379)	Mouse	WB(1/1,000)
Cyclin B1	abcam	ab2949	Rabbit	WB(1/1,000)
Cyclin D1	Cell signaling	92G2(#2978)	Rabbit	WB(1/1,000)
Cyclin E1	abcam	ab88259	Rabbit	WB(1/1,000)
p-p53^{S15}	Cell signaling	D4S1H(#12571)	Rabbit	WB(1/1,000)
Cleaved Caspase-3	Cell signaling	5A1E(#9664)	Rabbit	WB(1/1,000)
Myc-tag	Cell signaling	9B11(#2276)	Mouse	WB(1/10,000)
GFP	Roche	Clones(7.1 and 13.1)	Mouse	WB(1/1,000)

Supplementary information
Koyano *et al.*,

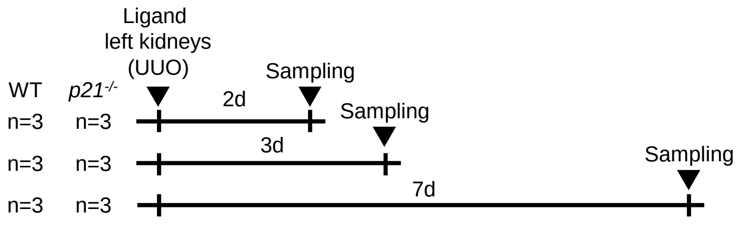

Figure S1. Schematic outline of unilateral ureter obstruction (UUO)

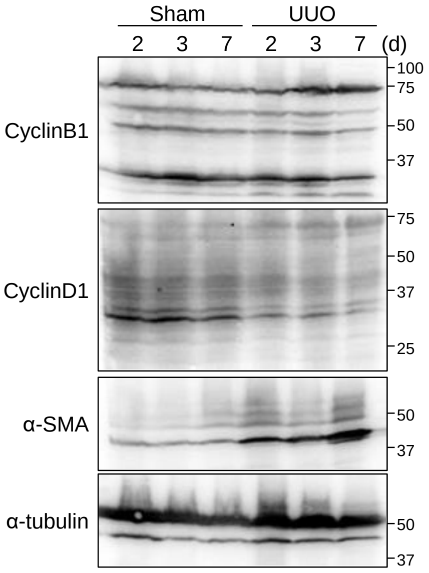

Figure S2. Protein levels of CyclinB1 and CyclinD1
Immunoblotting of tissue lysates from indicated samples. The sample are another set of Figure 2F. The molecular weight (kda) indicated in right sides of panels.

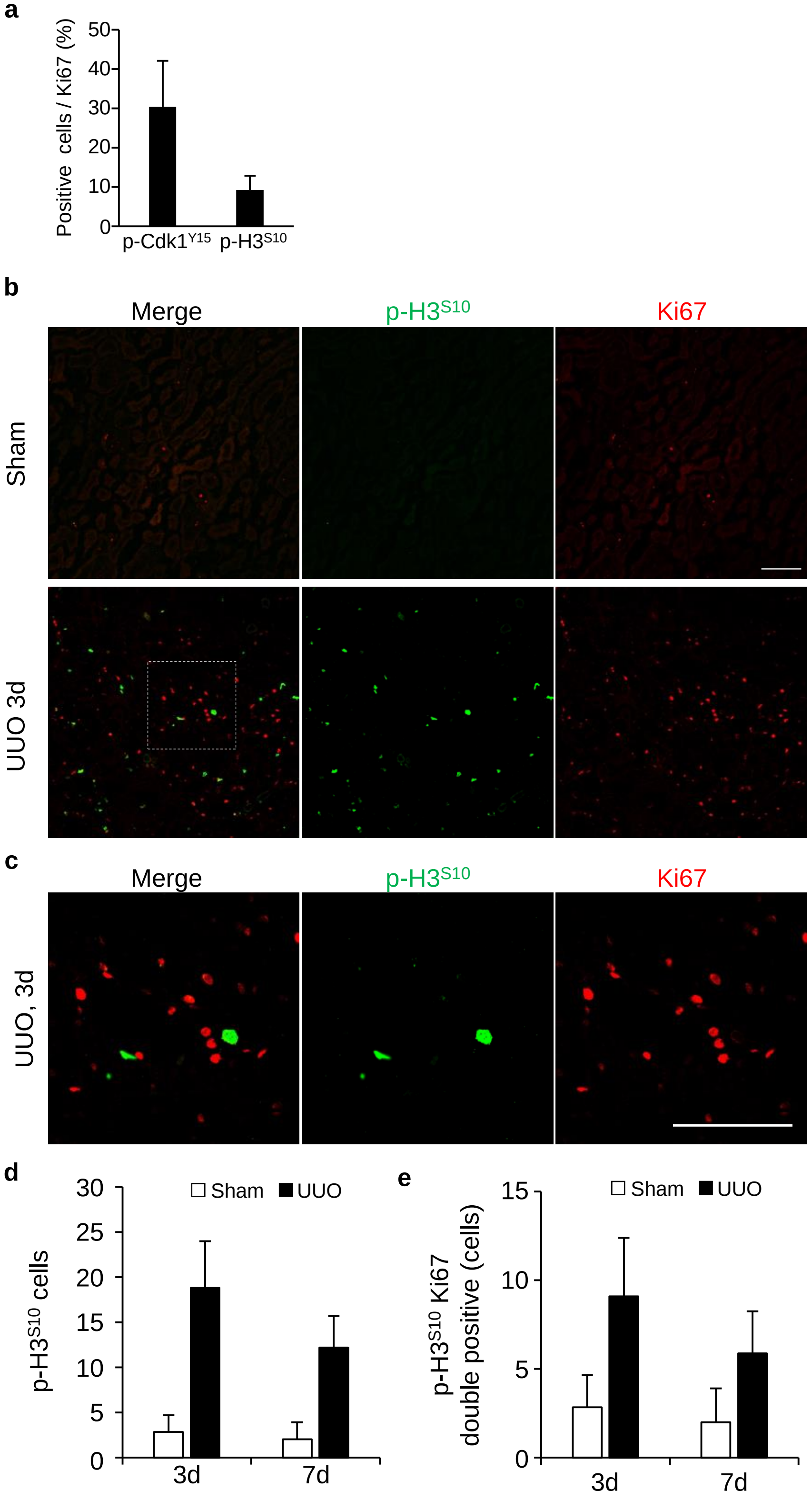

Figure S3. The image of double staining with p-H3^{S10} and Ki67

(a) A ratio of p-Cdk1^{Y15} or p-Histone H3^{S10} positive cells merged with Ki67 positive cells. **(b** and **c)** Co-immunostaining with Anti-p-H3^{S10} (green) and Ki67 (red). Cropped area is enlarged in (c). **(d)** Quantification of the number of p-H3^{S10} positive cells. Each staining were performed using different slices of the same sampling date. **(e)** The number of p-Histone H3^{S10} positive cells merged with Ki67 positive cells. *Bars*, 100µm. Data are as given averages ± SD.

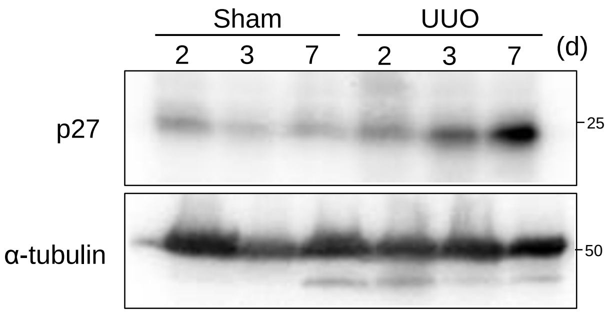

Figure S4. Protein levels of p27 after the injury
Immunoblotting of tissue lysates from indicated samples.
The molecular weight (kda) indicated in right sides of panels.

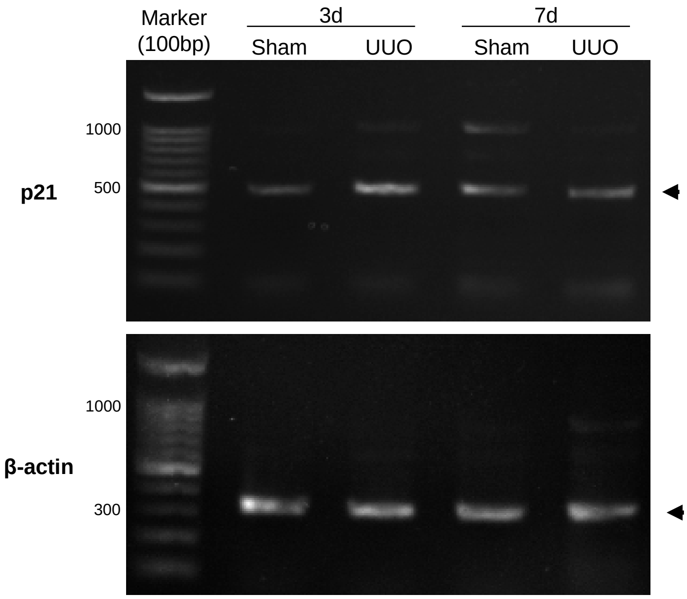

Figure S5. Expression level of p21 in mice after the injury

Total RNAs were isolated from the indicated kidneys. cDNAs for RT-PCR were synthesized from the total RNAs (ReverTraAce qPCR RT kit, TOYOBO). PCR was performed with using following primer pairs, p21 (Fw; 5'-atgtccaatcctggtgat and Rv; 5'-tcagggttttctcttgca), β-actin (Fw; 5'-ttcccctccatcgtgggccgc and Rv; 5'-gatggctacgtacatggctgg). β-actin was used as internal control. PCR reactions were performed with KOD plus neo (TOYOBO). The reaction parameter was 98°C for 15sec, 63°C for 15sec and 68°C for 30sec; 35cycles.

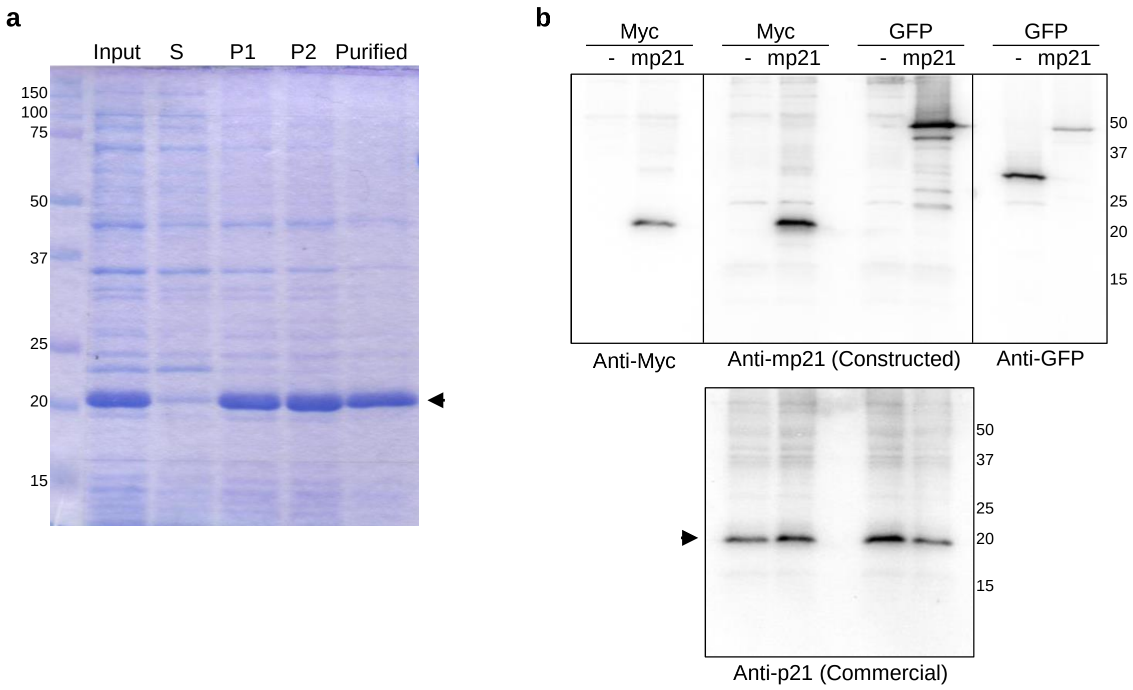

Figure S6. Construction of the monoclonal antibody against mp21
(a) SDS-PAGE analysis of the His-tagged mp21 protein. Arrow indicates the band of His-tagged mp21 . Proteins were stained with coomassie brilliant blue (CBB). S: Soluble fraction, P: Pellet. Purified protein was suspended in 8 M of urea. (**b**) Immunoblotting with the constructed monoclonal antibody. The cell lysates from cos-7 cells expressing Myc-mp21 or GFP-mp21. The indicated antibodies shown in upper panel were used, and the commercial antibody against p21 is used in bottom. Arrowhead indicates the endogenous p21. We could not detect mp21 bands (Myc-mp21 or GFP-mp21) by using the commercial antibody.

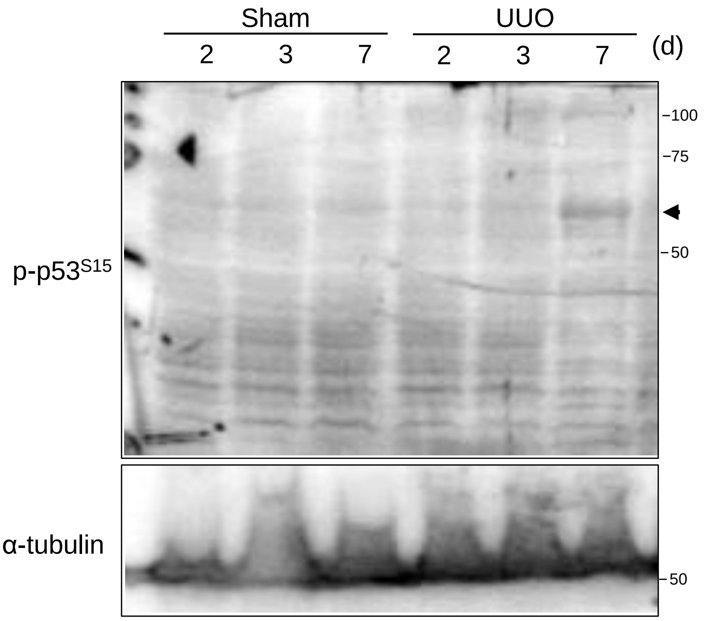

Figure S7. Levels of p-p53^{S15}
Immunoblotting of tissue lysates from indicated samples. The sample are another set of Figure 5. The molecular weight (kDa) indicated in right sides of panels.

Supplementary information
Koyano *et al.*,

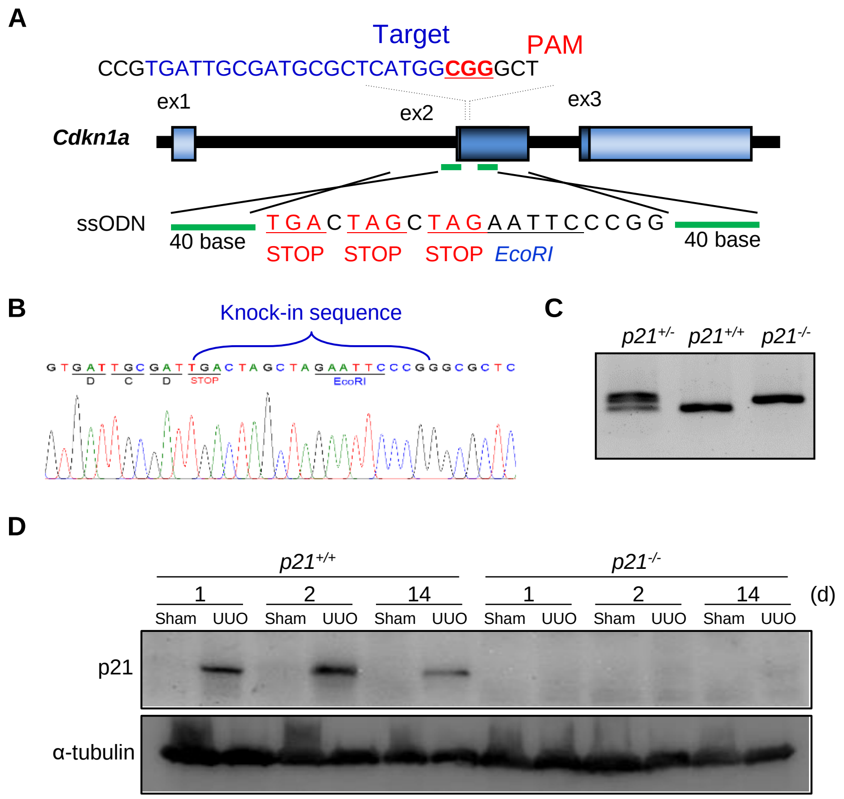

Figure S8. Construction of the *p21* deficiency mice

(a) The strategy for the construction of *p21* deficiency mice (*p21*$^{-/-}$). **(b)** Genome sequence of *p21*$^{-/-}$. **(c)** The gel image of PCR products amplified from indicated genotypes. **(d)** Immunoblotting with the mp21 antibody of tissue lysates.

Supplementary information
Koyano *et al.*,

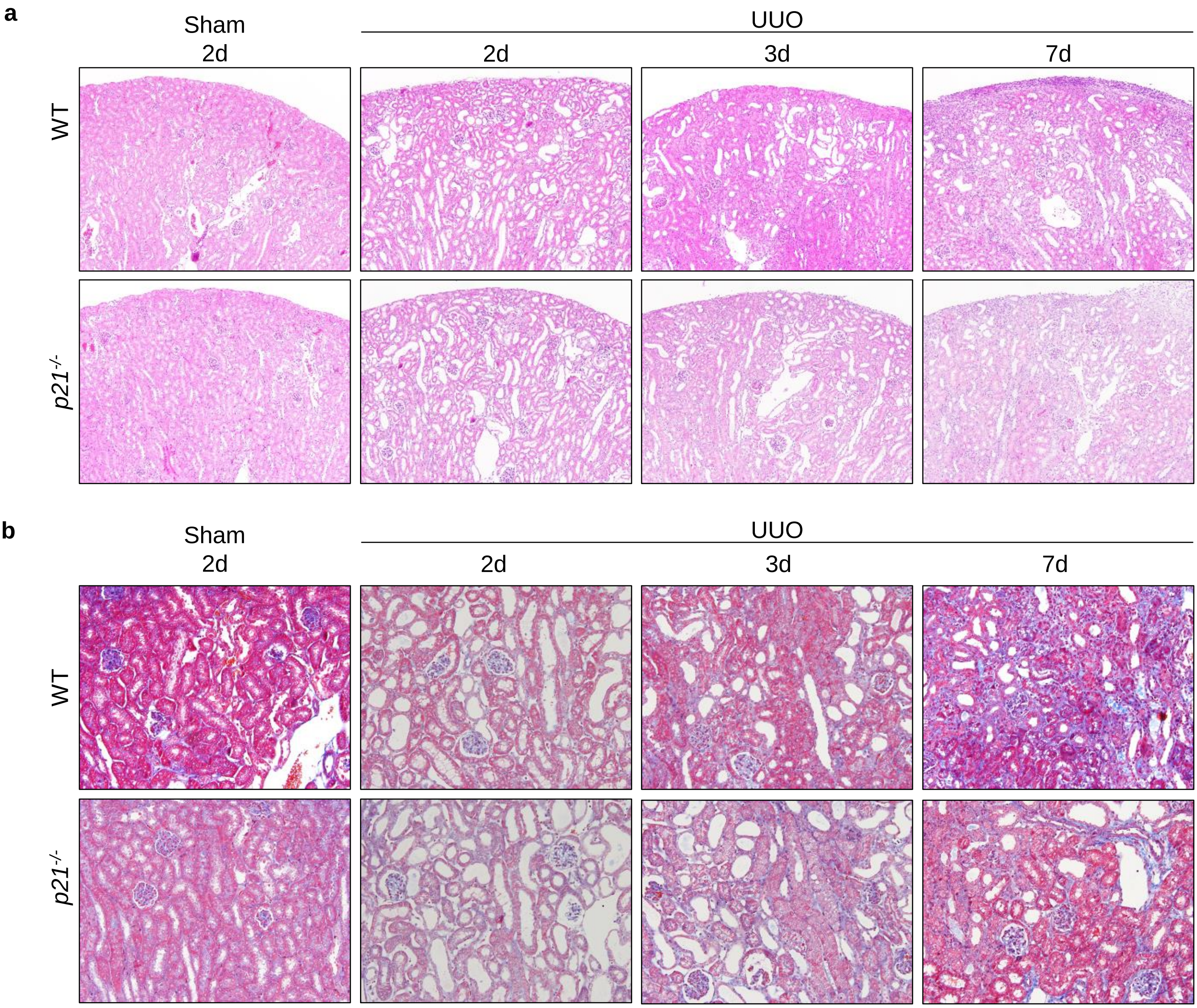

Figure S9. Histological staining of kidneys after UUO in *p21* deficiency mice
(a) Staining of Hematoxylin and Eosin (HE). The images were taken at x100 magnification. **(b)** Masson's trichrome staining. Fibrosis stained in blue. The images were taken at x200 magnification.

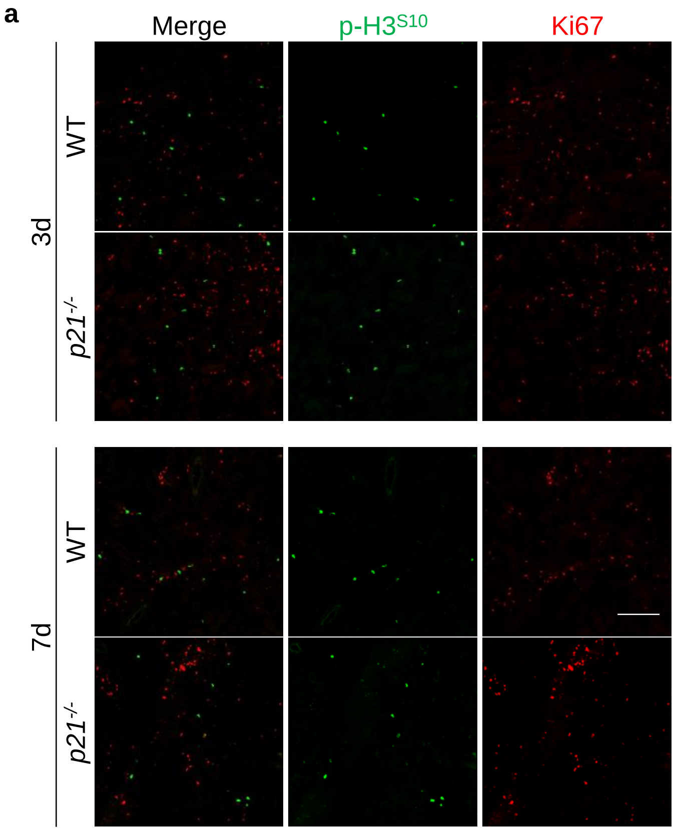

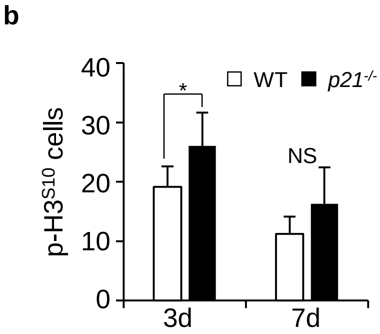

Figure S10. The number of p-H3S10 was slightly increased in *p21* deficient mice
(a) Co-immunostaining with Anti-p-H3S10 (green) and Anti-Ki67 (red). *Bar*, 100μm. **(b)** The number of p-$H3^{S10}$ positive cells. Data are as given averages ± SD; $*P < 0.05$; NS, Not Significant (n=3 for each time points in each genotypes, two-tailed unpaired Student's t-test).

Supplementary information
Koyano *et al.*,

Fig. 2a

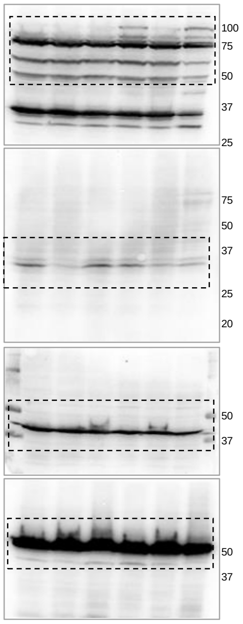

Fig. 2b

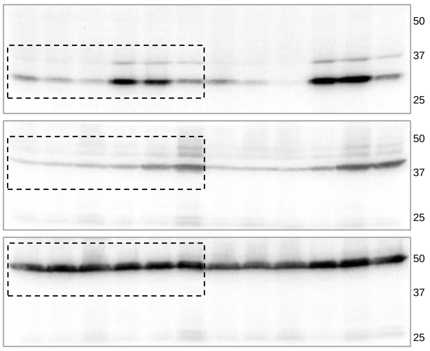

Fig. 5

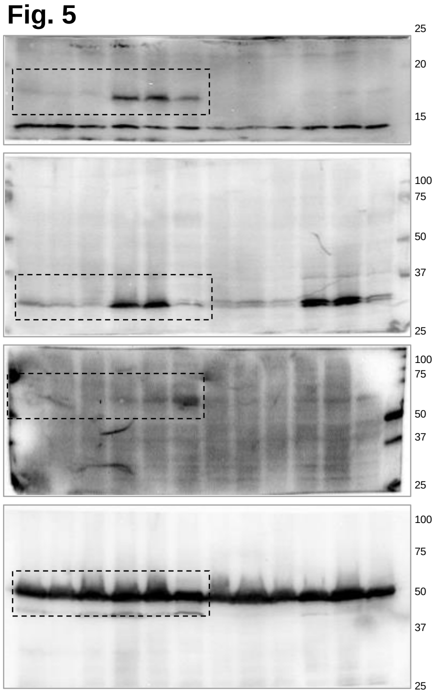

Fig. 3a

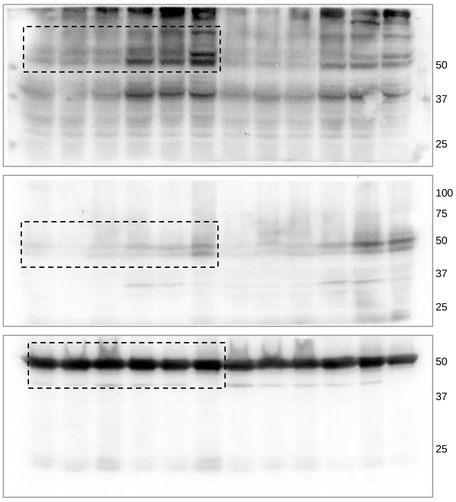

Fig. 3d

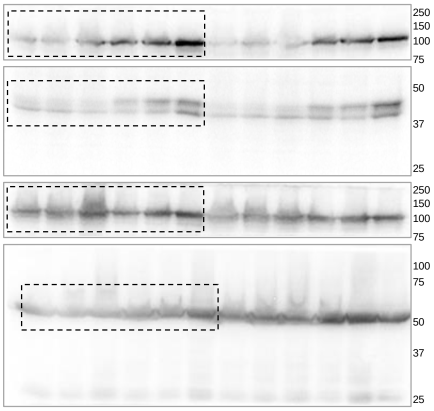

Fig. 4b

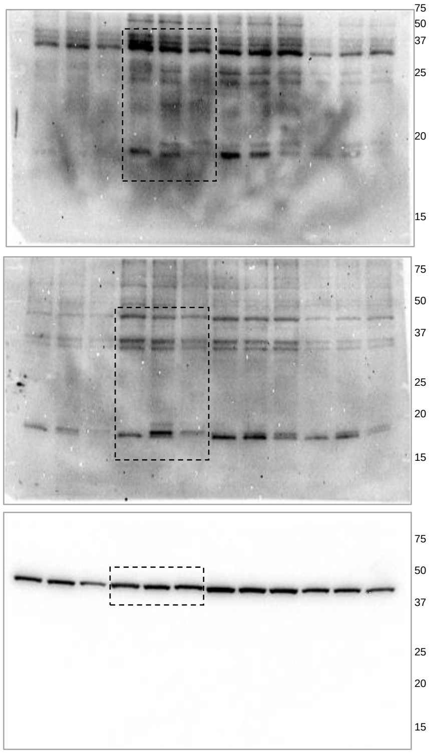

Fig. 3c

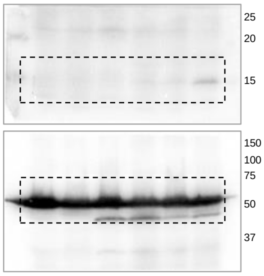

Fig. 4a

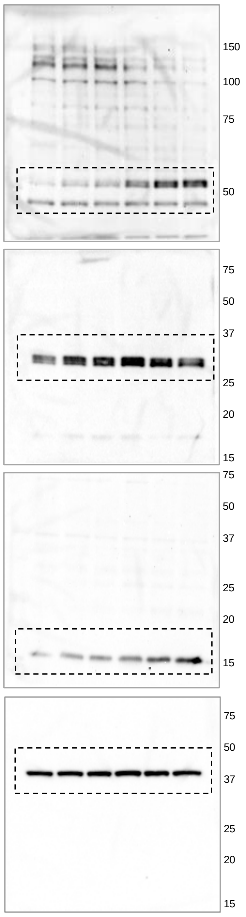

Fig. 6e

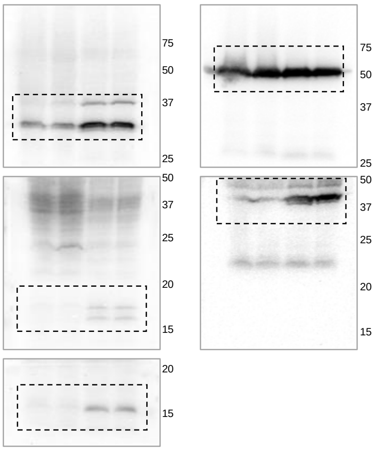

Figure S11. Full scans of images
The positions of markers (kDa) are shown in right-hand sides of the images.
